# Concurrent Diagnosis of Acute Myeloid Leukemia and COVID-19: A Management Challenge

**DOI:** 10.7759/cureus.9629

**Published:** 2020-08-09

**Authors:** Abdul Moiz Khan, Zainub Ajmal, Mihir Raval, Ellis Tobin

**Affiliations:** 1 Internal Medicine, Albany Medical Center, Albany, USA; 2 Hematology and Oncology, Albany Medical Center, Albany, USA; 3 Infectious Disease, Upstate Infectious Diseases Associates, Albany, USA

**Keywords:** covid 19, novel corona virus, corona virus disease 2019, acute myeloblastic leukemia, acute myeloid leukemia (aml), remdesivir, convalescent plasma therapy

## Abstract

The emergence of coronavirus disease 2019 (COVID-19) has created new challenges in the management of serious diseases. We describe a 41-year-old male who presented with fever, watery diarrhea, and epistaxis. Initial workup revealed pancytopenia with >50% blasts on the peripheral smear raising suspicion of acute myeloid leukemia (AML) (later confirmed by bone marrow biopsy as AML with myelodysplasia-related changes) and a positive polymerase chain reaction (PCR) for severe acute respiratory syndrome coronavirus 2 (SARS-CoV-2). Given the extraordinary risk, he was treated with remdesivir and convalescent plasma for COVID-19. On admission day 8, repeat PCR for SARS-CoV-2 returned negative and the patient was deemed stable for chemotherapy. Therefore, induction was done with liposomal daunorubicin and cytarabine. However, he did not respond to the therapy and was started on re-induction therapy with decitabine and venetoclax. In our discussion, we review the current principles of treatment of patients with concurrent COVID-19 and AML.

## Introduction

The emergence of coronavirus disease 2019 (COVID-19) caused by severe acute respiratory syndrome coronavirus 2 (SARS-CoV-2) has brought forth new challenges in the management of serious diseases including hematological malignancies. There is a dearth of data on the prognosis and optimal management of patients with concomitant COVID-19 and hematological malignancies [1,2]. In general, some studies suggest that cancer patients may have a higher rate of COVID-19 infection, severe illness, rapid development of severe symptoms and mortality [1-4]. While it is of utmost importance to minimize the risk of COVID-19 in the immunocompromised population, patients require intensive monitoring and modified treatment strategies once the infection occurs.

We describe a patient with newly diagnosed acute myeloid leukemia (AML) and COVID-19, and discuss the current approach of management of such cases.

## Case presentation

A 41-year-old male with no significant past medical history presented with fever with rigors and chills, fatigue, myalgias, 4-5 episodes daily of watery diarrhea, diffuse abdominal pain and nausea for four days. He also reported one episode of coffee-ground emesis and multiple episodes of epistaxis within the last month. He had some exertional dyspnea but denied cough, chest pain, sore throat, rhinorrhea, anosmia or ageusia. He also denied any travel outside of New York state. On presentation, he had a temperature of a 101 F, tachycardia up to 125 beats/minute, blood pressure at 142/65 mmHg and oxygen saturation was 97% on room air. On physical examination, prominent conjunctival pallor and mild generalized abdominal tenderness were noted. Lungs were clear to auscultation.

Initial workup revealed severe pancytopenia with >50% blasts on the peripheral smear raising strong suspicion of acute leukemia, and positive polymerase chain reaction (PCR) for SARS-CoV-2 on nasopharyngeal swab. The patient emergently received intravenous (IV) cefepime and vancomycin for empirical antimicrobial coverage for neutropenic sepsis as well as packed red blood cells and platelet transfusions.

Investigations

The results of the initial investigations are provided in Table 1.

**Table 1:** Initial investigations

Laboratory test	Result	Reference range
White blood cells	2.8 x 10^3^ cells/μL	3.4 x 10^3^-10.8 x 10^3^ cells/μL
Absolute neutrophil count	0.1 x 10^3 ^cells/μL	1.5 x 10^3^-5.2 x 10^3^ cells/μL
Absolute lymphocyte count	0.3 x 10^3 ^cells/μL	1.1 x 10^3^ -3.9 x 10^3^ cells/μL
Hemoglobin	5.9 g/dL	11.1-15.9 g/dL
Hematocrit	16.4%	34-46.6%
Platelets	12 x 10^3^ cells/μL	150 x 10^3^-450 x 10^3^ cells/μL
Prothrombin time	16.3 seconds	9.8-11.8 seconds
International normalized ratio	1.4	0.83-1.14
Fibrinogen	654 mg/dL	172-483 mg/dL
D. dimer	10.56 mg/L	<0.5 mg/L
Lactate dehydrogenase	527 IU/L	90-225 IU/L
Haptoglobin	235.3 mg/dL	24-234 mg/dL
Erythrocyte sedimentation rate	41 mm/hr	0-32 mm/hr
C-reactive protein	392.4 mg/L	<8 mg/L
Procalcitonin	27.84 ng/mL	<0.5 ng/mL
Ferritin	1591 ng/mL	24-336 ng/mL
Troponin	0.2 ng/mL	0-0.04 ng/mL
Bicarbonate	19 mmol/L	21-30 mmol/L
Blood urea nitrogen	29.3 mg/dL	7-22 mg/dL
Creatinine	1.8 mg/dL	0.8-1.4 mg/dL
Estimated glomerular filtration rate	49.38	>60
Uric acid	6.1 mg/dL	3.6-8 mg/dL

**Figure 1:**
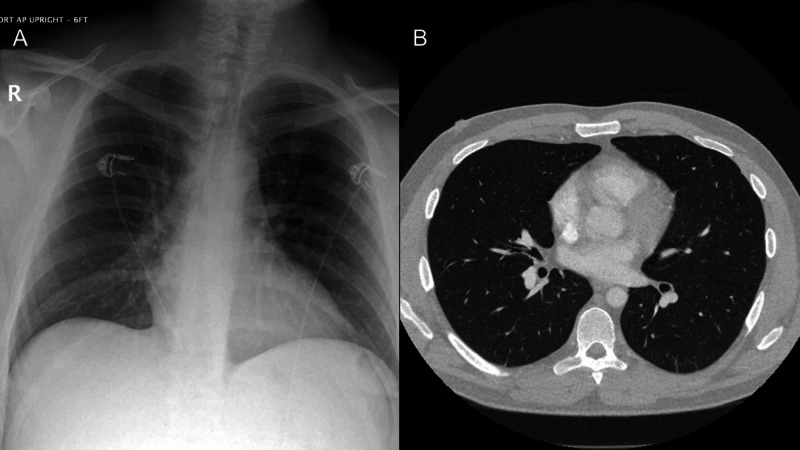
(A) Chest X-ray and (B) chest CT are both negative for any findings of interstitial or lobar pneumonia

X-ray and computed tomography (CT) scan of the chest were negative for interstitial or lobar pneumonia (Figure 1). CT scan of the abdomen and pelvis showed only mild thickening of small bowel loops.

Clostridium difficile toxin gene was detected by PCR in the stool sample. However, enzyme immunoassay (EIA) was negative for the toxin. Urine culture, blood cultures and stool culture were negative.

Peripheral blood flow cytometry revealed 58% large blasts, positive for cluster of differentiation (CD)117, human leukocyte antigen-DR (HLA-DR), CD34, CD13, CD22 (dim), CD33 (dim, partial), CD56, and CD45 (moderate), and negative for CD11b, CD14, myeloperoxidase (MPO), CD3, CD4, CD5, CD7, CD10, CD19 and CD20.

Bone marrow aspirate was markedly hypercellular with blast population constituting 69% of marrow cellularity on 500-cell-count differential. Multiparametric flow cytometry analysis of bone marrow aspirate detected 68% large blasts, positive for CD117, HLA-DR, CD34, CD13, CD22 (dim), CD56, and CD45 (moderate), had a partial dim expression of CD7, CD11b, and CD33, and were negative for CD14, MPO, CD3, CD4, CD5, CD10, CD19, CD20, cyCD3 and surface light chains.

Bone marrow biopsy showed hypercellular marrow consisting of sheets of blasts, representing 70%-75% of the overall marrow cellularity. The blasts were highlighted by CD34, CD117, and MPO, and were negative for E-cadherin. FMS-like tyrosine kinase 3 (FLT3) internal tandem duplication (ITD) and FLT3 tyrosine kinase domain mutations were not detected. AML fluorescence in-situ hybridization (FISH) showed 11q+, 8q+, and 6p-, myelodysplastic syndrome (MDS) FISH showed 5q- and 8+. Chromosome analysis revealed an abnormal male karyotype with all cells (20/20 cells) exhibiting a complex composite hyperdiploidy karyotype with multiple structural and numerical abnormalities including trisomy 1, structural rearrangement of 5q that may lead to loss of 5q, trisomy 8, and trisomy 21. Next-generation sequencing detected a pathogenic frameshift genomic alteration in the TP53 gene (c.636delT; p.R213Dfs*34). Overall, these findings were consistent with AML with myelodysplasia-related changes (AML-MRC), stratified in the poor/adverse risk category given the 5q deletion, complex karyotype, and TP53 mutation.

Further treatment and outcome

The patient was managed under the close supervision of hematology and infectious disease specialists. He was treated with remdesivir (first dose 200 mg IV, followed by 100 mg IV daily for four more days) and convalescent plasma for COVID-19. Chemotherapy for AML was delayed to manage COVID-19 appropriately and ensure clinical stability first.

Our patient also tested positive for Clostridium difficile gene but not for toxin. However, given the presence of watery diarrhea, possibility of a false negative result and the overall complexity of the case, oral vancomycin, and IV metronidazole were started. He was also given one dose of IV bezlotoxumab (1000 mg) to reduce the risk of recurrence of Clostridium difficile infection (CDI).

Fortunately, the patient remained stable from the perspective of respiratory system. He also had an appreciable improvement in diarrhea. Packed red blood cell and platelet transfusions were administered as indicated for the anemia and thrombocytopenia. Antimicrobial therapy with cefepime for neutropenic fever, acyclovir for antiviral, and voriconazole for antifungal prophylaxis was continued throughout. Symptomatic care with antipyretics and antiemetics was provided and the patient was closely monitored for DIC and tumor lysis syndrome. 

On admission day 8, SARS-CoV-2 real-time PCR was repeated which returned negative. Considering the relative stability and the reassuring negative COVID-19 test, induction therapy for AML was initiated with liposomal daunorubicin 44 mg/m2 and cytarabine 100 mg/m2 on days 1, 3, and 5.

Hospital course after the induction chemotherapy was complicated by recurrent fever and prominent mucositis. Antibiotics were escalated empirically to meropenem and briefly with the addition of IV vancomycin. Patient continued to have profound pancytopenia and >50% blasts on the blood smear. Peripheral blood flow cytometry done 14 days after the induction revealed 55% myeloid blasts suggesting a lack of response to therapy and obviating the need of repeat bone marrow biopsy. Therefore, re-induction therapy with decitabine and venetoclax was started. Prognosis remains poor overall given the adverse risk AML, unresponsiveness to initial chemotherapy and complications of neutropenia.

## Discussion

The concurrence of AML and COVID-19 presents an extraordinary challenge of treating two potentially life-threatening diseases at the same time. The National Cancer Research Institute (NCRI) AML working group and other expert reviews recommend screening all the patients with AML for SARS-CoV-2 prior to initiation of therapy regardless of symptoms [3,5]. Although the induction for newly diagnosed AML is often done on an emergent basis, a reasonable delay in order to test a symptomatic patient for COVID-19, or to provide appropriate management for the COVID-19 infection may be a prudent approach [6]. Therefore, the NCRI AML working group recommends delaying the therapy for AML, if possible, until symptoms resolve and PCR becomes negative [5]. In our patient, we followed this approach of instituting therapy for COVID-19 and obtaining a negative PCR for SARS-CoV-2 prior to induction for the AML. However, a careful determination should be made on a case-by-case basis under expert guidance.

Accurate prognostication of AML with COVID-19 is difficult given the paucity of studies, small sample sizes, and multiple potential confounders. Ferrara et al. have described ten COVID-19 patients with AML, seven of which developed rapid worsening of respiratory function, seven required modifications in the hematological treatment, five died after a median of eight days and death was COVID-19 related in all cases [7]. In addition, Núñez-Torrón et al. have described four patients with AML and COVID-19, three of which developed refractory acute respiratory distress syndrome (ARDS) and eventually died [8]. Of note, none of the patients in these two studies received remdesivir or COVID-19 convalescent plasma. Our patient did not develop a severe respiratory illness from COVID-19 as evident clinically and further substantiated by the imaging. Although it would be hard to assert with certainty, the prompt use of remdesivir and convalescent plasma may have benefitted our patient in that regard.

The choice of treatment regimen for AML depends on the cytogenetic profile and other patient factors. However, general considerations to mitigate the risk of adverse outcomes in AML patients with COVID-19 include reducing the dose of myelosuppressive agents like cytarabine, incorporating less immunosuppressive drugs like venetoclax and azacitidine, decreasing the number of cycles of chemotherapy in select cases, using granulocyte colony stimulating factor (G-CSF) for neutropenia, adding prophylactic antimicrobial agents, and monitoring for drug interactions that may result in adverse effects like QTc prolongation [5,6,9]. Allogeneic stem cell transplant should still be pursued if clinically indicated and safe, as for other patients [5,6].

Principles of COVID-19 treatment have been evolving constantly. As of June 16, 2020, there were no Food and Drug Administration (FDA) USA approved drugs for COVID-19 treatment, though various interventions are available through FDA emergency use authorization. Remdesivir has shown benefit in shortening the time to recovery in adults hospitalized with COVID-19 and evidence of lower respiratory tract infection [10]. The National Institute of Health (NIH) USA guidelines recommend remdesivir for the treatment of COVID-19 in hospitalized patients with oxygen saturation ≤94% on ambient air, and those who require supplemental oxygen, mechanical ventilation or extracorporeal membrane oxygenation (ECMO). Recommended treatment duration is five days in general, and 10 days for patients on mechanical ventilation or ECMO, or who do not have clinical improvement in five days [11]. Furthermore, some studies have reviewed COVID-19 convalescent plasma as an effective therapeutic option [12]. However, the NIH guidelines comment that there is insufficient data to recommend either for or against its use for the treatment of COVID-19 [13]. Although our patient did not have a prominent respiratory illness from COVID-19, the immunosuppression from AML itself and the anticipated chemotherapy posed an exceptional risk of deterioration. Therefore, we pursued with treating our patient with both remdesivir and convalescent plasma.

Another interesting point in our case is the utilization of bezlotoxumab, a monoclonal antibody directed against C. difficile toxin B, indicated as an adjunct treatment for CDI and associated with a significant reduction in the risk of recurrent CDI over 12 weeks in patients with risk factors for recurrence [14,15]. Significant benefits have been demonstrated in patients with one or more of the risk factors that include age >65, history of CDI, immunocompromise, or severe CDI [14]. The presence of severe immunocompromise made a strong case for using bezlotoxumab in our patient.

## Conclusions

In the current circumstances, all the patients with AML should be screened for COVID-19 prior to initiation of therapy regardless of symptoms. Chemotherapy for AML may be reasonably delayed in order to provide appropriate management for the COVID-19 infection. Remdesivir and convalescent plasma may be beneficial in some patients although there are no FDA USA approved drugs for COVID-19 to date.
